# IMP3 as a cytoplasmic biomarker for early serous tubal carcinogenesis

**DOI:** 10.1186/s13046-014-0060-2

**Published:** 2014-07-20

**Authors:** Yiying Wang, Lingmin Li, Yue Wang, Zeng Yuan, Wenjing Zhang, Kenneth D Hatch, Wenxin Zheng

**Affiliations:** 1 Department of Obstetrics and Gynecology, Henan Provincial People’s Hospital, Zhengzhou, China; 2 Department of Pathology, University of Arizona College of Medicine, Tucson, AZ; 3 Department of Pathology, Shanxi Medical University, Taiyuan, China; 4 Department of Obstetrics and Gynecology, Qilu Hospital, Shandong University, Jinan, China; 5 Department of Obstetrics and Gynecology, University of Arizona, Tucson, AZ; 6 Arizona Cancer Center, University of Arizona, Tucson, AZ

**Keywords:** IMP3, Serous tubal intraepithelial carcinoma, STIC, p53 signature, High-grade serous carcinoma

## Abstract

**Background:**

Serous tubal intraepithelial carcinoma (STIC) and the p53 signature in tubal mucosa have been supported to be precursor lesions in high-grade serous carcinoma (HGSC) of the fallopian tube, ovary, and peritoneum. It remains critical to find biomarkers for precursor lesions in order to detect HGSCs efficiently. IMP3 is an oncoprotein that has been explored in human malignancies. No studies have specifically addressed the expression of IMP3 in precursor or early lesions of HGSC. The main purposes of this study are to evaluate if IMP3 plays any role in the process of pelvic serous carcinogenesis by examining its expression in HGSC precursor lesions, to examine the relationship between IMP3 and p53 in those precursor lesions, and to check if IMP3 can be used as a biomarker for early diagnosis.

**Methods:**

Immunohistochemistry for IMP3 and p53 was performed and evaluated in 48 HGSCs with STIC, 62 HGSCs without STIC, and 60 benign cases as negative controls. Sections of fallopian tubes with or without STIC , as well as cancers within the ovaries, were studied. IMP3 signature was defined as strong IMP3 cytoplasmic staining in 10 or more consecutive benign-looking tubal epithelial cells. The relationship between IMP3 and p53 overexpression was examined.

**Results:**

In the 48 HGSC patients with STIC, IMP3 was positive in 46% of STIC lesions and had a similar positive rate in the invasive components of HGSC. IMP3 was also expressed in normal appearing tubal epithelia (IMP3 signature) in 15 (31%) of 48 HGSC cases with STIC and 10 (16%) of 62 cases without STIC. In contrast, no single IMP3 signature was found in the benign control group. Concordant expression of IMP3 and p53 signatures in the STIC group was found in up to one-third of the cases. There were also five (10%) STIC cases with positive IMP3 and negative p53.

**Conclusions:**

We conclude that IMP3 may be involved in the process and progression of pelvic HGSC and may serve as a complimentary biomarker in diagnosing STIC.

## Introduction

Pelvic serous cancer (PSC), including mainly high-grade serous carcinoma (HGSC) that involves the primary sites of the ovary, the fallopian tube, and the peritoneum, is the most common and lethal type of müllerian malignancy, comprising more than 70% of all malignancies from these organs [[1]–[3]]. Effective management of this disease has been hampered because up to 90% of HGSC in patients are discovered in the advanced stages. Therefore, investigators have emphasized the importance of understanding the early phases of this fatal disease, such as precancerous or intraepithelial lesions, in order to find an effective method for early detection [[4]]. The accumulated studies in the past decade have revealed that the sources of pelvic HGSCs are mainly derived from the distal fallopian tube rather than the ovary or the peritoneum [[3],[5]–[11]]. A noninvasive carcinoma of the fallopian tube, designated as ‘serous tubal intraepithelial carcinoma’ (STIC), is found in up to 60% of pelvic HGSC patients [[12]]. STIC, mainly localized in the distal tube, is considered as the morphologically identifiable precursor lesion for HGSC since the cancer cells remain in the tubal epithelial layer. However, via an unclear molecular mechanism, the cancer cells of STIC are able to detach from the tubal mucosa and “implant” on the ovarian and peritoneal surfaces and grow into the status of carcinomatosis within the pelvis or abdominal cavity. Therefore, elucidation of the early phase of pelvic high-grade serous carcinogenesis will shed light on early detection and cancer prevention.

Although tubal high-grade serous carcinogenesis remains elucidated, alteration of *TP53* is a well-known gene that plays a key role for cancer initiation and development [[13]]. This was supported by the finding of p53 signatures, defined as intense p53 protein overexpression in the normal looking tubal epithelia [[9]]. This particular stretch of the tubal epithelia is most commonly seen in the tubal fimbria, mainly in tubal secretory cells, and *TP53* gene mutations have been found in more than 50% of the cells with p53 signatures [[9]]. Because of this critical molecular change, tubal epithelia with p53 signatures are now considered as latent precancer for HGSC [[3],[14],[15]]. STICs, as well as invasive HGSCs, have been found to harbor *TP53* mutations in over 90% of cases and the majority of them stain strongly and diffusely with the p53 antibody [[9],[16]]. Based on these observations, we believe that tubal HGSC follows a stepwise developmental model and that p53 serves as an important biomarker for those serous lesions in the entire cancer developmental process. However, as we all know, carcinogenesis typically involves more than a single gene. In addition, there are some significant portions of early serous tubal epithelial lesions that are negative for p53 immunostaining. Therefore, other biomarkers found in this setting will be useful for early diagnosis.

IMP3, an oncoprotein, is a member of insulin-like growth factor II mRNA binding proteins, also known as IGF2BP3 [[17],[18]]. IMP3 is epigenetically silenced soon after birth, with little or no detectable protein in normal adult tissues [[19]] except in placentas and gonads [[20]]. Re-expression of IMP3 is observed in a series of human malignancies, including ovarian, endometrial, and cervical cancers, correlating with increased risk of metastases and decreased survival [[19],[21]–[23]]. Not only overexpressed in those invasive cancers, IMP3 has also been considered as a marker of preinvasive lesions within the cervix and the endometrium [[22],[24]]. IMP3 has also been used as a prognostic marker for all ovarian cancer patients in our routine pathology practice, during which IMP3 overexpression was sometimes observed in normal appearing tubal mucosa as well as in STIC cases. Such findings prompted us to examine the following questions: 1) whether IMP3 expression is involved in the early process of tubal HGSC development, 2) if IMP3 can be used as a diagnostic marker for STIC, and 3) the relationship between IMP3 and p53 in the process of tubal high-grade serous carcinogenesis.

## Materials and methods

### Case collection

A total of 170 identified cases were pulled from pathology files of the University of Arizona Medical Center. The institutional review board approved the study. There were three groups of patients in the study: HGSC with STIC (n = 48), where these HGSCs were classified as tubal primary since STIC was identified in tubal fimbriated ends; HGSC without STIC (n = 62); and the positive control, which included ovarian HGSC patients without identifiable STIC. Pathologic examination of the fallopian tubes revealed that 53 cases had invasive cancer foci in the tubal wall or paratubal soft tissue, but no STICs were identified. All of the cancer patients had no history or either chemotherapy or radiation therapy prior to the surgical staging. Family history of ovarian cancer and personal history of breast cancer were collected, but BRCA mutation status was not available. In addition to the tissue samples obtained from the above HGSC patients, we also studied tubal tissues from a group of patients with benign gynecologic diseases (n = 60) as negative controls. These patients had no evidence of any malignancy and came to the hospital for total hysterectomies and bilateral salpingo-oophorectomy because of leiomyomata, endometriosis, or uterine prolapse. The ages ranged from 42 to 75 with an average age of 61.5 years.

### Tissue handling

All of the fallopian tube samples were handled using SEE-FIM protocol [[3],[25]] for those cancer patients since this is the routine procedure in UMC. Fallopian tubes from benign control cases were processed by embedding all fimbriated ends similar to cancer patients with additional representative 2 cross sections of the ampulla as described previously [[10]].

All tissues were fixed in 10% buffered formalin and processed routinely for paraffin embedding. Five-micron sections for IHC were cut and placed on Super Plus slides (Fisher Scientific, Pittsburgh, PA) before sectioning each specimen for hematoxylin and eosin staining in order for them to be examined microscopically for diagnostic confirmation.

### Morphologic analysis

The secretory and ciliated cells within the tubal mucosa were readily identifiable under the light microscopy. To further confirm the cell type, we stained the tubal sections with PAX8 (marker for secretory cells) and tubulin (marker for ciliated cells). STIC is a noninvasive carcinoma confined to the epithelial cells of fimbriae and is characterized by significant cytologic atypia and/or atypical intraepithelial proliferation. The histologic diagnoses of STIC were made based on criteria described previously [[26]].

### Immunohistochemical analysis

The IMP3 antibody (L523S) was provided by Dako (Carpinteria, CA), which was a mouse monoclonal antibody (MAb) specific for the IMP3/KOC antigen. Immunohistochemical stains were performed on 5-um tissue sections from representative blocks using the purified mouse anti-IMP3 antibody and the standard avidin-biotin-complex technique as described previously [[27]–[29]]. Representative sections of endometrial serous carcinoma served as positive controls for the IMP3 antibody [[29]]. Negative controls were performed by replacing the primary antibody with nonimmune IgG. All slides were reviewed independently by two investigators (YW and WZ). The percentage of neoplastic cells and nonneoplastic tissues that showed dark brown cytoplasmic staining was recorded. The intensity of the IHC staining was recorded as absent, weak, moderate, or strong. IMP3 overexpression in STIC or PSC was defined as >10% of the stained cancer cells with strong intensity of the cytoplasmic staining. IMP3 signature was defined as strong cytoplasmic IMP3 staining in 10 or more benign appearing tubal epithelial cells. PAX8 has been considered as a müllerian epithelial marker identifying tubal secretory as described previously [[10]].

Immunohistochemical analysis for p53 protein expression was performed as described previously. Assessment of immunohistochemical results for p53 was based on distinct nuclear staining. For cancer cases, positive staining was defined by staining more than 75% of the cancer nuclei with at least a moderate degree of staining intensity. Occasional cytoplasmic p53 staining was considered as negative.

### Statistical analysis

The mean values and standard errors were calculated, and the paired t test was used by PROC MEANS in the SAS system. P values less than 0.05 were considered statistically significant.

## Results

### Patient characterization

This study examined IMP3 expression in the fallopian tubes of patients from the following three groups: HGSC with STIC, HGSC without STIC, and benign controls. The HGSC with STIC group included 48 patients who were identified by STIC in the fallopian tubes. Patients’ ages at surgery in this group ranged from 38 to 81 years with an average age of 57.2 years, which was about 10 years younger than that of the HGSC without STIC group (36 to 89 years with average of 67.1 years) (P < 0.005). The clinicopathologic characteristics of the two HGSC groups are summarized in Table 1.

**Table 1:** Clinicopathologic features of high-grade serous carcinoma with and without STICHGSC: high-grade serous carcinoma; STIC: serous tubal intraepithelial carcinoma

	**HGSC w/ STIC (n = 48)**	**HGSC w/o STIC (n = 62)**	***P***
	**No. (%) patients**		
**Age (y) mean ± SD**	57.2 ± 2.78	67.1 ± 2.32	< 0.005
≦40	4	2	
41-50	9	6	
51-60	18	11	
61-70	10	22	
> 70	7	21	
**STIC locations**			
Left tube	12		
Right tube	29		
Bilateral tubes	7		
**Invasive locations^**			
Left	3	4	
Right	5	6	
Bilateral	37	52	> 0.05
**Cancer size (cm) mean ± SD**			
Fallopian tube	0.55 ± 0.21	2.66 ± 0.72	< 0.05
Ovary	3.42 ± 0.52	4.35 ± 0.64	> 0.05
**Stage**			
I	4	0	< 0.05
II	5	3	> 0.05
III	39	51	> 0.05
IV	0	8	< 0.05
**Breast cancer history**	8	7	
**Family history**	12	12	
**Prophylactic BSO**	5	0	

^indicating the adnexal location of those invasive cancers. Among the 48 STIC patients, 3 showed STIC only without invasive component. w/: with; w/o: without.

For those cases without gross lesions in the fallopian tube, the lesion size was measured microscopically.

### IMP3 in normal looking tubal epithelia

**Figure 1:**
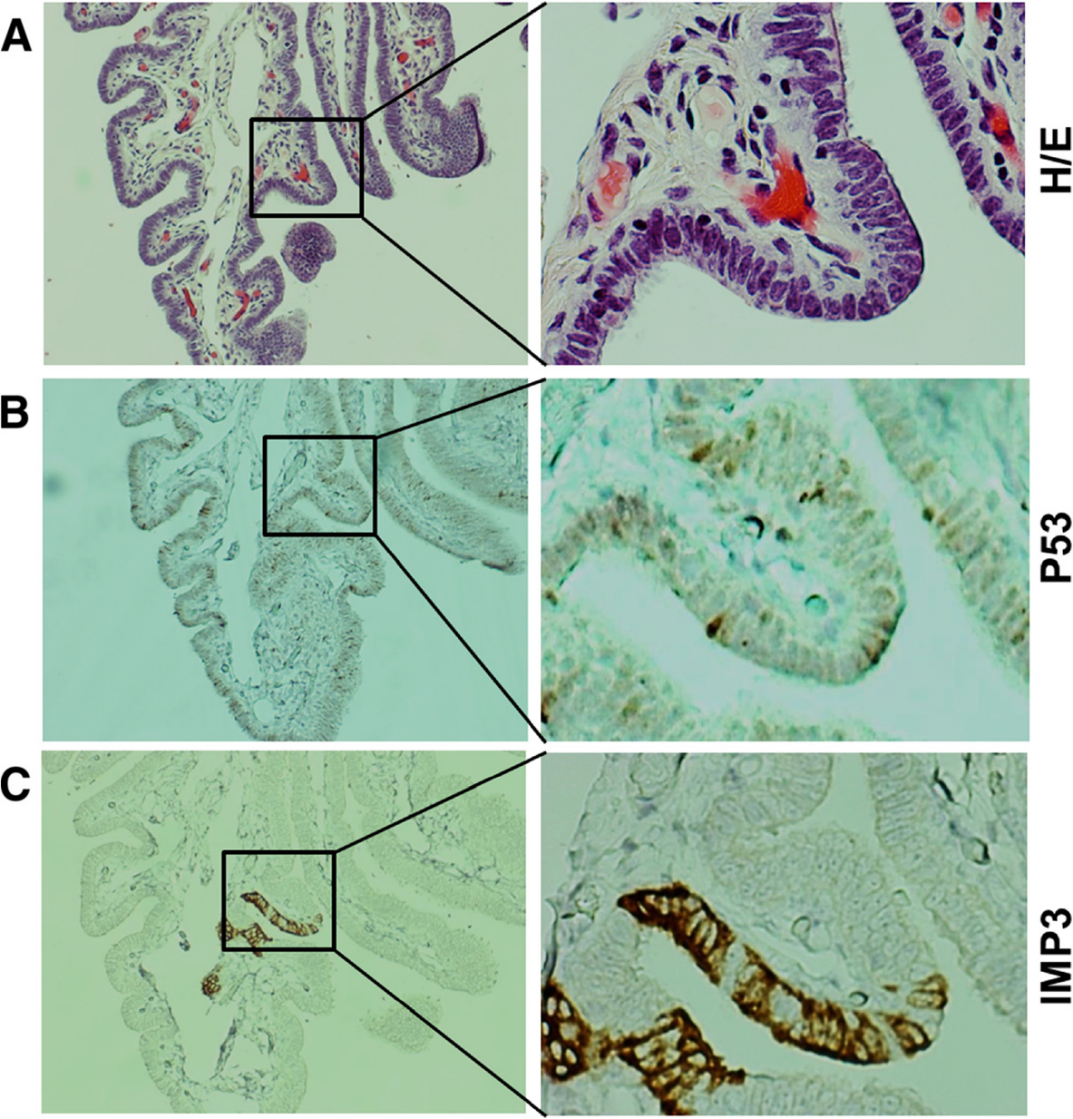
**Differential expression of IMP3 and p53 in normal tubal epithelial cells. A**. H/E staining of normal epithelia of the fallopian tube. **B**. P53 was occasionally positive in some normal-looking secretory cells of the fallopian tube, which typically representing wild type *TP*53. **C**. IMP3 was strongly expressed in focal area of secretory cells in the fallopian tube, barely in ciliated cells in the only one case of the benign group. Ciliated cells could be appreciated by cilia on the left of panel **A**. Original magnifications: Left panel 40x, right panel 200x.

To evaluate if IMP3 was overexpressed in normal looking tubal epithelial cells, we examined IMP3 expression in sections of the fallopian tube from the two study groups (STIC group, n = 48, and HGSC without STIC, n = 62) and one control group (n = 60). The benign control fallopian tubes were obtained from patients without any gynecologic malignancy. In the benign control group, IMP3 was found to be weakly and occasionally moderately immunoreactive in 1 of the 60 cases in less than 1% of the tubal epithelial cells. Immunoreactivity for IMP3 was present mainly in secretory cells and barely in ciliated cells (Figure 1). In contrast, IMP3 immunoreactivity was significantly increased in the normal looking tubal epithelia in both study groups (see the results of IMP3 signature below).

PAX8 and p53 were also examined in the parallel sections of the fallopian tubes from the control group. Immunoreactivity for PAX8 was found only in secretory cells (data not shown), consistent with our previously reported studies [[10],[30]]. The immunoreactivity for p53 was not observed in the normal fallopian tubes from patients with benign gynecologic diseases, but it was found in the study groups (see the results of p53 signature below).

### The relationship between IMP3 and p53 signatures

**Figure 2:**
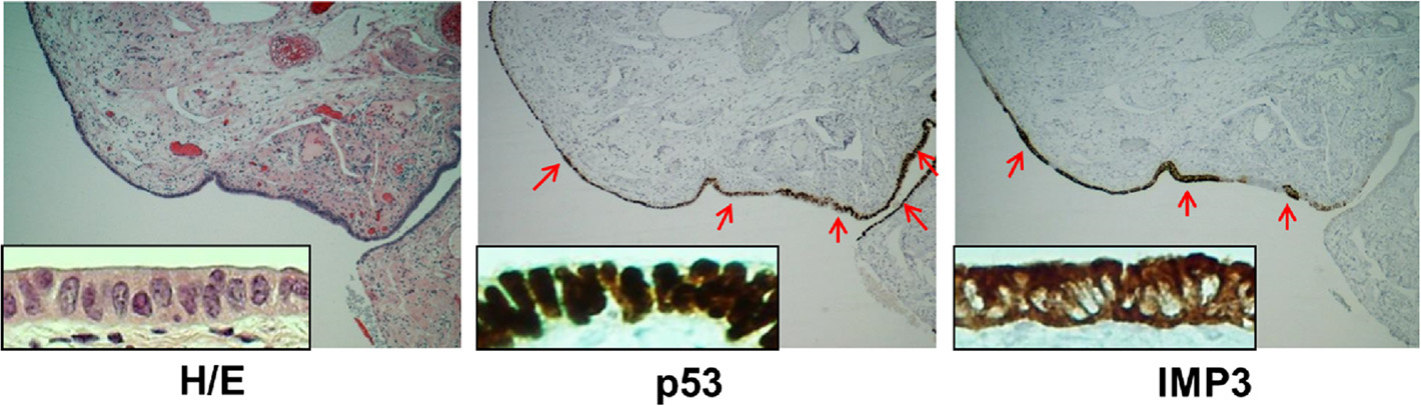
**IMP3 and p53 signatures in tubal epithelia from a high-risk patient.** Photographs illustrated examples of normal-looking epithelia in fimbria with strong immunoreactivity for IMP3 and p53 (40x). A closer view of the IMP3 and p53 signatures was shown in inserts (200x) of the panel. Immunoreactivity for IMP3 and p53 were identified in 2 different sites indicated by red arrows in the same fallopian tube. Apparently, the majority of the IMP3 and p53 signatures were overlapped in this particular stretch of the tubal epithelia.

IMP3 signature was defined as the criteria similar to those of the p53 signature previously described [[31]]: the presence of moderate-to-strong immunoreactivity for IMP3 in at least 10 consecutive secretory cells in the fallopian tube showing no more than moderate cytologic atypia and no intraepithelial proliferation. There were no IMP3 signatures found in the 60 benign control fallopian tubal samples. However, 15 (31%) of 48 patients with STIC and 10 (16%) of 62 cancer patients without STIC showed IMP3 signatures, respectively. Among the total of 25 cancer cases with IMP3 signature, nine showed p53 signatures in the same group of the cells, eight were located in the different regions of the tubal mucosa, and eight were negative for p53. A total of 38 p53 signatures were found in cancer group with 20 (53%) in the STIC patients and 18 (47%) in the HGSC without STIC group. No p53 signatures were found in the benign control group. The representative pictures of IMP3 signatures in relationship with p53 signatures are present in Figure 2 and summarized in Table 2.

**Table 2:** The relationship between IMP3 and p53 signatures^ in tubal epithelia

**Case group (No.)**	**# IMP3 signatures (%)**	**# p53 signatures (%)**	**# Conc (%)**	**# Discord (%)**	**# Indep (%)**
Benign (60)	0	0			
w/STIC (48)	15 (31)	20 (53)	5(33)	4(27)	6(40)
w/oSTIC(62)	10 (16)	18 (47)	4(40)	4(40)	2(20)

^IMP3 or p53 signature is defined by either moderate or strong immunostainings in benign appearing tubal epithelia. Compared to the benign and cancer cases without STIC, the number of IMP3 signature was significantly higher in the tubal epithelia of the cases with STIC with p values of < 0.0001 and < 0.05, respectively.

#Conc: the number of concordance; #Discord: the number of discordance; #Indep: the number of independent signatures of IMP3 and p53. STIC: serous tubal intraepithelial carcinoma. w/: with; w/o: without. The concordance, discordance, and independent rate were calculated from the IMP3 signature data after comparing the cases with p53 signature. The reverse relationship was not evaluated in this study.

### IMP3 and p53 Expression in STIC

**Figure 3:**
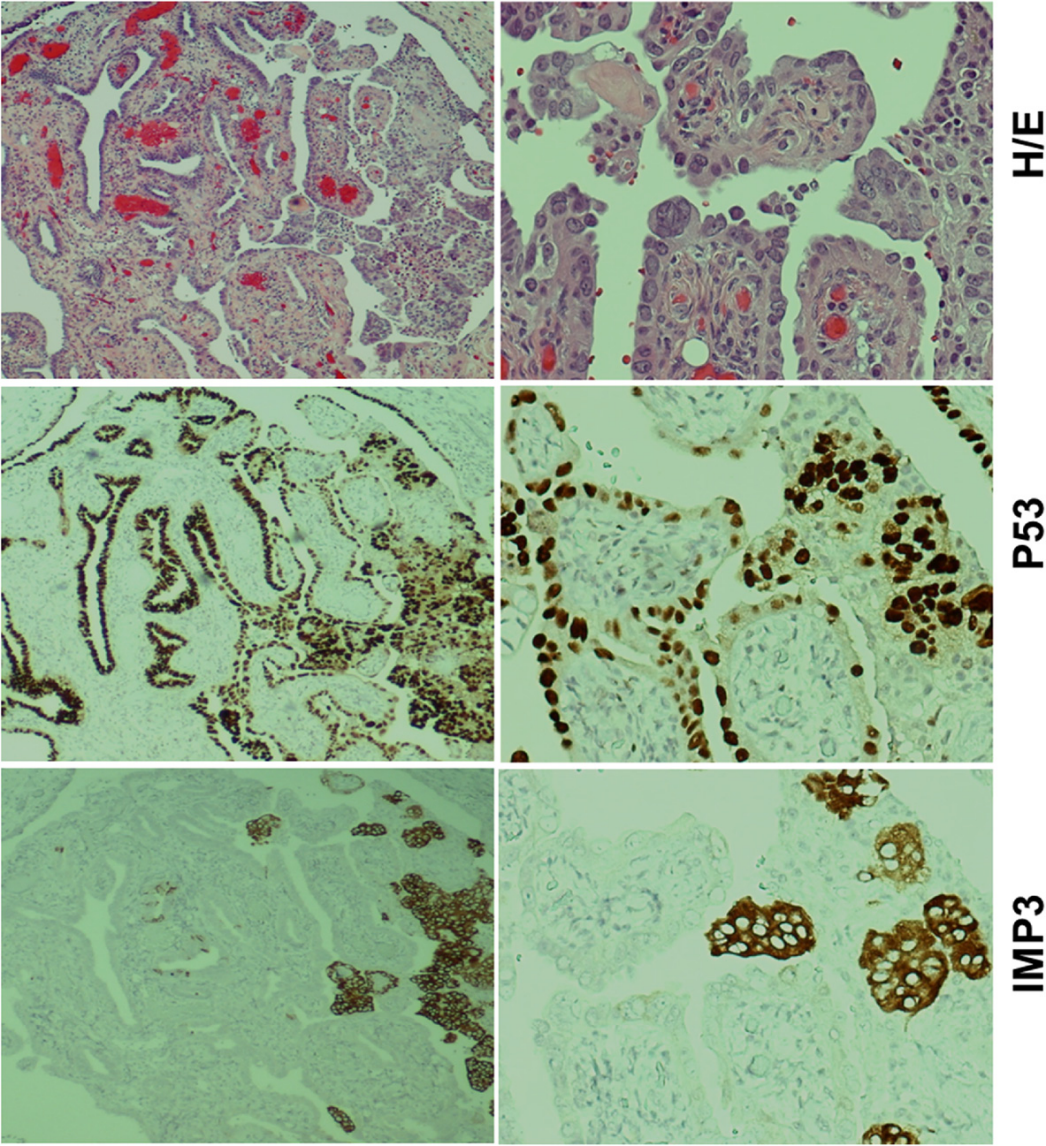
**IMP3 and p53 overexpression in serous tubal intraepithelial carcinoma (STIC).** STIC (top panel) was strongly positive for both p53 (mid panel) and IMP3 (low panel). Apparently, this case showed more intraepithelial cancer cells were positive for p53 than those of IMP3. However, some of the neoplastic cells were positive for both p53 and IMP3 (right side of the mid and low panels). Original magnifications: left panel, 40x; right panel, 200x.

The positive IMP3 overexpression was defined as more than 10% of the target cells showing at least moderate intensity staining in the cytoplasm [[29]], while p53 positivity was defined as more than 75% of intense nuclei staining of the target cells [[32]]. Among the 48 patients with areas of STIC we studied, we observed positive IMP3 in 22 (46%) and p53 overexpression in 40 (83%) cases, respectively. The positive expression of IMP3 in STIC ranged from 15% to 100% cancer cells with an average of 45.5%. Among the 22 IMP3 positive cases in STIC, 17 (77%) were positive and five (23%) were negative for p53 staining. Within the same 48 STIC patients, eight (17%) cases showed negative expression for both IMP3 and p53. The representative pictures of IMP3 and p53 for STIC and the corresponding data are presented in Figure 3 and Table 3.

**Table 3:** IMP3 and p53 immunoreactivity in STIC and invasive HGSC

			**Invasive HGSC of ovary**			
	**STIC**		**W/ STIC**		**W/O STIC**	
	**No. (%) cases**	***P***	**No. (%) cases**	***P***	**No. (%) cases**	***P***
**IMP3+**	22 (46)		20 (42)		25 (40)	
**IMP3-**	26 (54)	0.82	28 (58)	0.56	37 (60)	0.71
**p53+**	40 (83)		42 (88)		53 (85)	
**p53-**	8 (17)	< 0.01	6 (12)	< 0.01	9 (15)	< 0.01
**IMP3+/p53+**	17 (35)		17 (35)		19 (31)	
**IMP3+/p53-**	5 (10)	<0.05	3 (6)	<0.05	7 (11**)**	<0.05
**IMP3-/p53+**	18 (38)		20 (42)		28 (45)	
**IMP3-/p53-**	8 (17)	0.26	8 (17)	0.16	9 (15)	0.08

Note: The total number of patients with STIC (serous tubal intraepithelial carcinoma) cases studied was 48, while the number of invasive ovarian HGSC (high-grade serous carcinoma) without STIC was 62. The percentage of positive or negative IMP3 with the relationship to p53 staining results was calculated by the total IMP3 positive or negative cases. The *p* values listed in the table represented the comparisons within the same group of patients showing different status of IMP3 and/or p53.

### IMP3 and p53 Expression in HGSC

We further examined the expression of IMP3 and p53 in the invasive components of HGSC in both study groups (STIC group, n = 48, and HGSC without STIC, n = 62). Within the STIC group, the staining results for IMP3 and p53 in the invasive cancer areas were very similar to those found in the areas of STIC (Figure 3) with the exception of the two cases. These two cases showed positive IMP3 and negative p53 in STIC, but they were reversed (negative IMP3 and positive p53) in the invasive component. Interestingly, eight (20%) cases with negative expression for both IMP3 and p53 in STIC were also negative in the corresponding invasive areas (Table 3).

**Figure 4:**
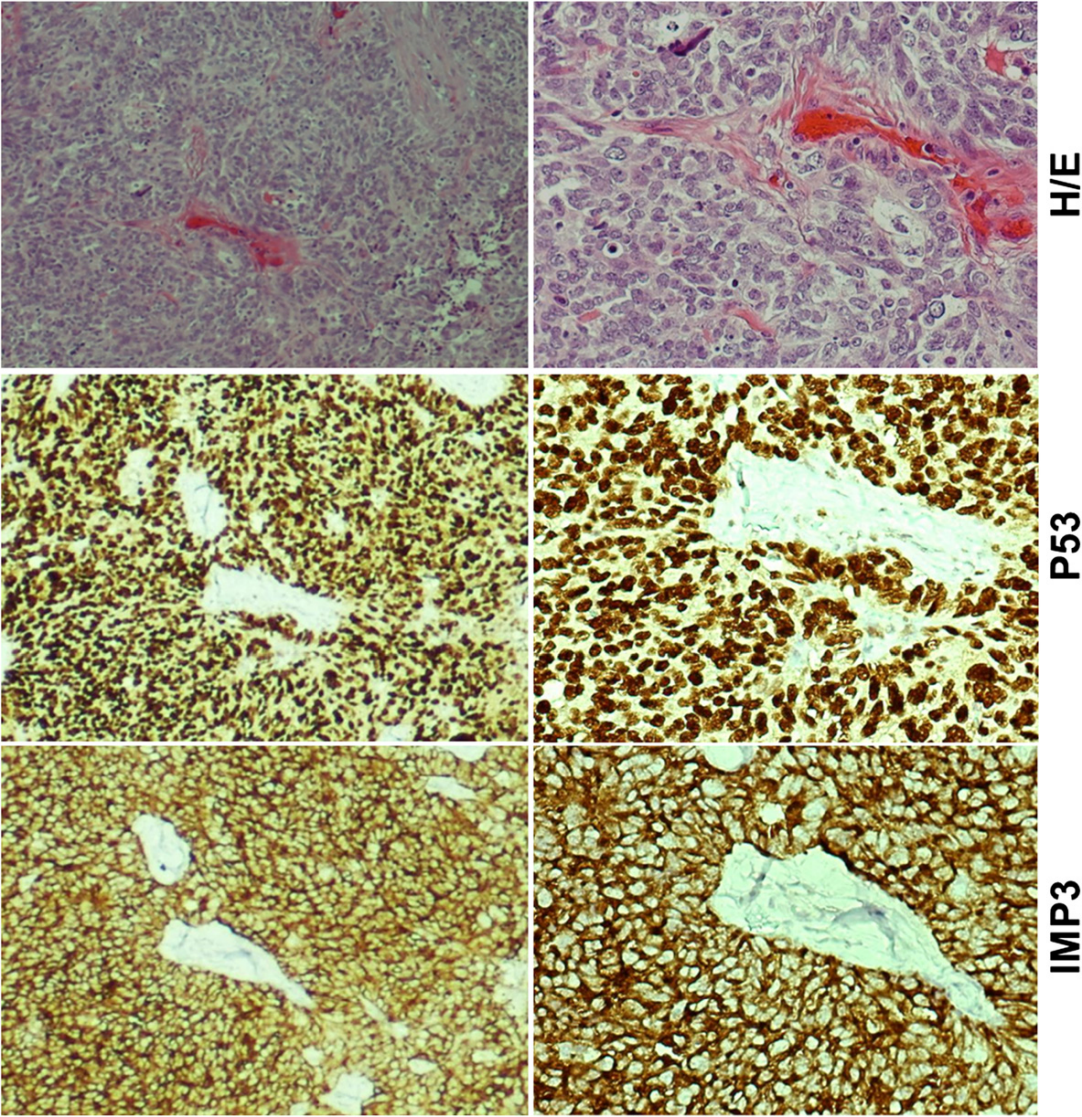
**IMP3 and p53 overexpression in invasive component of high-grade serous carcinoma (HGSC).** Example of invasive HGSC (top panel) showed positive for both p53 (mid panel) and IMP3 (low panel). Original magnifications: left panel, 40x; right panel, 200x.

In the patients of HGSC without STIC group, the overall staining results for these two markers were also similar to those cancer cells in the STIC group (Figure 4). The detailed results are presented in Table 3.

## Discussion

Although IMP3 expression, which is associated with tumor growth, progression, and unfavorable prognosis, has been explored in a number of human malignancies, only two studies on immunohistochemical analysis for IMP3 in ovarian cancers have been published. Kobel et al. demonstrated IMP3 expression in 86% of mucinous tumors, in about half of clear-cell and high-grade serous carcinomas, and in 27% of endometrioid cancers [[19]]. Noske et al. detected expression of IMP3 in 32 (47%) of 68 ovarian carcinomas but did not report their findings according to various histologic types [[33]]. However, no studies have been addressed regarding the IMP3 expression in precursor or early lesions of HGSC of either tubal or “ovarian” origins.

In this study, we have shown that IMP3 signatures, defined as strong positive cytoplasmic staining in more than 10 benign appearing consecutive tubal epithelia, were found in 15 (31%) of the 48 cases with STIC. This is in contrast to the benign control group, which showed no single IMP3 signature, found in 60 studied cases (p < 0.0001). Interestingly, the tubal IMP3 signature rate was also significantly higher than those in 10 (16%) of the 62 cancer cases without STIC (p < 0.05). Additionally, concordance expression of IMP3 and p53 signatures in the STIC group was found in up to one-third of the cases, while the remaining was either discordant or independent (Table 2). Overall, our findings suggest that *IMP3* aberration may play a different role than *TP53* in the initial phase of tubal serous carcinogenesis.

Another important finding of this study is that IMP3 overexpression was frequently expressed (46%) in patients with STIC who had invasive HGSC in the ovary. Although this positive rate is less than the p53 positivity (83%) in the same group of cases, the concordant positive staining for both IMP3 and p53 biomarkers was found in 35% of the STIC cases. More interestingly, there were five (10%) STIC cases showing positive IMP3 staining but were negative for p53 overexpression. These findings suggest that IMP3 staining may aid the diagnosis of STIC, particularly in those cases with negative p53 staining.

Although the majority of HGSC in the pelvis is currently classified into tubal primary, particularly when STIC is present [[3],[7],[34]], the cancers mainly involving the ovary but without STIC are, by convention, still classified as ovarian primary. Our finding of similar IMP3 expression rate (Table 3) as well as similar clinicopathologic presentations in HGSC with or without STIC supports that HGSC without finding STIC is also likely arising in the fallopian tube [[3]]. One of the common reasons for not finding STIC in those ovarian HGSCs is likely due to limited tubal samples examined under microscopy or advanced cancer growth obliterating the tubal fimbria.

Based on the findings discussed above, we conclude that IMP3 may involve the initial process of pelvic high-grade serous carcinogenesis and pelvic serous cancer progression. IMP3 may serve as a complimentary biomarker to aid the diagnosis of STIC, particularly when it is negative for p53 immunostaining. However, since this study is mainly on the immunostaining level, detailed molecular mechanism studies are needed to address if tubal epithelia with IMP3 signatures actually represent a latent precancer and if it has a synergistic role in facilitating cancer development with *TP53*. Other studies such as the risk of IMP3 signatures in cancer prediction and overexpression of IMP3 in HGSC in relation to patient survival and response to adjuvant therapies are also pertinent in the near future.

## Abbreviations

PSC: Pelvic serous carcinoma

HGSC: High-grade serous carcinoma

STIC: Serous tubal intraepithelial carcinoma

SCE: Secretory cell expansion

SCOUTs: Secretory cell outgrowths

## Competing interests

The authors declare no conflict of interest.

## Authors’ contributions

YYW, KDH and WXZ conceived the study design and experiments. YYW, LL, ZY, and WJZ carried out experiments and data analysis. YYW, LL, YW, ZY, WJZ, KDH, WXZ wrote the manuscript. All authors were involved in editing and approving the final manuscript.
